# Investigation of the Interaction Mechanism of Perfluoroalkyl Carboxylic Acids with Human Serum Albumin by Spectroscopic Methods

**DOI:** 10.3390/ijerph17041319

**Published:** 2020-02-18

**Authors:** Huilun Chen, Qianyu Wang, Yanping Cai, Rongfang Yuan, Fei Wang, Beihai Zhou

**Affiliations:** School of Energy and Environmental Engineering, and Beijing Key Laboratory of Resource-oriented Treatment of Industrial Pollutants, University of Science and Technology Beijing, 30 Xueyuan Road, Haidian District, Beijing 100083, China; s20180190@xs.ustb.edu.cn (Q.W.); cyp38948@foxmail.com (Y.C.); yuanrongfang@ustb.edu.cn (R.Y.); wangfei6699@aliyun.com (F.W.)

**Keywords:** perfluoroalkyl carboxylic acids (PFCAs), human serum albumin (HSA), fluorescence, toxicity, carbon chain length, molecular docking

## Abstract

Perfluoroalkyl carboxylic acids (PFCAs) are some of the most significant pollutants in human serum, and are reported to be potentially toxic to humans. In this study, the binding mechanism of PFCAs with different carbon lengths to human serum albumin (HSA) was studied at the molecular level by means of fluorescence spectroscopy under simulated physiological conditions and molecular modeling. Fluorescence data indicate that PFCAs with a longer carbon chain have a stronger fluorescence quenching ability. Perfluorobutanoic acid (PFBA) and perfluorohexanoic acid (PFHxA) had little effect on HSA. Fluorescence quenching of HSA by perfluorooctanoic acid (PFOA) and perfluorodecanoic acid (PFDA) was a static process that formed a PFCA–HSA complex. Electrostatic interactions were the main intermolecular forces between PFOA and HSA, while hydrogen bonding and van der Waals interactions played important roles in the combination of PFDA and HSA. In fact, the binding of PFDA to HSA was stronger than that of PFOA as supported by fluorescence quenching and molecular docking. In addition, infrared spectroscopy demonstrated that the binding of PFOA/PFDA resulted in a sharp decrease in the β-sheet and α-helix conformations of HSA. Our results indicated that the carbon chain length of PFCAs had a great impact on its binding affinity, and that PFCAs with longer carbon chains bound more strongly.

## 1. Introduction

Perfluoroalkyl carboxylic acids (PFCAs), such as perfluorooctanoic acid (PFOA), have been widely used in the manufacture of global consumer goods, including non-stick kitchen utensils, surface treatment agents, and surfactants [1,2]. Since 2000, their persistence, bioaccumulation, and toxicity have attracted global attention [3,4]. Low concentrations of PFCAs are detected in environmental media samples, wildlife, and even in human serum [5,6,7,8,9]. Most interestingly, PFCAs are mainly accumulated in plasma, liver, and kidney due to their protein affinity [10,11]. In recent years, many studies have been conducted to assess the potential impact of PFOA on human health [7,12,13,14]. However, owing to the lack of toxicological data for other PFCAs, the risk assessment of environmental pollution caused by PFCAs is still very limited.

The study of PFCA–protein interactions was first conducted in the 1950s with the objective of protecting bovine serum albumin (BSA) from denaturation by interaction with PFOA and subsequent precipitation [15]. Since organofluorine chemicals have been reported in human plasma [16], and PFCAs have been regarded as global pollutants, the interactions between PFCAs and proteins have once again captured the attention of researchers. So far, PFCA–protein binding properties have been used primarily to study the toxicity of PFCAs through direct or competitive binding tests. Many studies have used direct binding tests to discover the effects of interactions on protein structures and functions and the potential mechanisms [10,17,18,19,20]. Besides toxicity assessment, the binding of PFCAs to proteins was also studied to understand bioaccumulation, biotransformation, and elimination of PFCAs [3,21,22].

Serum albumin is the main binding target of PFCAs in vivo [23]. Two studies previously described by Han et al. [11], and Jones et al. [24] confirmed the interaction of PFCAs with serum albumin. Chen and Guo [23] studied the binding of five perfluorinated compounds to human serum albumin (HSA) via site-specific fluorescence, and found that these chemicals bind to HSA with similar affinity to fatty acids at the same sites. MacManus-Spencer et al. [25] compared three experimental methods to examine the binding of PFCAs to serum albumin, and pointed out that although fluorescence is an indirect method, it can provide a more comprehensive description of the nature of the interaction. Qin et al. [26] investigated the effect of PFCAs on BSA through fluorescence spectroscopy, and demonstrated that PFCAs did affect the structure of BSA, and that PFCAs with a longer carbon chain length had greater toxicity at lower concentrations. Our previous work investigated the effect of functional groups (carboxylate or sulfonate headgroup) of perfluorinated compounds on binding sites and binding affinities [27]. However, the interaction between PFCAs with different carbon chain length and transporters in aqueous media is still far from being fully understood, so this topic needs to be carefully explored from different perspectives.

Liu et al. [10] summarized the main methodologies for characterizing PFCA–protein binding, including separation methods, calorimetric techniques, surfactant nature-based methods, spectroscopy, mass spectrometry, surface plasmon resonance, and molecular docking. Fluorescence has proven to be a reasonable way to supply comprehensive quantitative and qualitative information about the PFCA–albumin complexation [25]. As a continuation of previous research [20,23,26], the goal of this paper was to systemically characterize the molecular mechanism of the PFCA–HSA complexation by means of fluorescence spectroscopy under simulated physiological conditions. Additionally, the effects of PFCAs with different carbon chain length on secondary structure changes of HSA were compared using FT-IR spectroscopy. Furthermore, the binding affinity of PFCAs on HSA was also interpreted by molecular docking. This study may help to understand the molecular mechanism of PFCAs’ toxicity on HSA.

## 2. Materials and Methods

### 2.1. Materials

All chemicals and reagents were of analytical grade, including fatty acid-free HSA (A1887, lyophilized powder, Sigma-Aldrich, St. Louis, MO, USA), perfluorobutanoic acid (PFBA, 98%, Sigma-Aldrich, St. Louis, MO, USA), perfluorohexanoic acid (PFHxA, 98%, Sigma-Aldrich, St. Louis, MO, USA), PFOA (98%, Chemical Industry, Tokyo, Japan), perfluorodecanoic acid (PFDA, 98%, Sigma-Aldrich, St. Louis, MO, USA). The stock solution of each PFCA was prepared in phosphate-buffered saline (PBS, pH 7.4 ± 0.1). HSA solution was freshly prepared in PBS (pH 7.4 ± 0.1) 15 min before use. In order to prevent PFCAs from adsorbing to glass surface, polypropylene containers were used.

### 2.2. Fluorescence Spectrometry Measurements

A Hitachi spectrofluorometer (Model F-2700, 1.0 cm quartz cell, Hitachi, Tyoko, Japan) equipped with a xenon lamp (150 W) and a thermostat was used to record fluorescence emission spectra at different temperatures (296, 303, and 310 K) in the 290–500 nm wavelength range. The widths of excitation slits and emission slits were both set to 5.0 nm. The excitation wavelength was set to 295 nm. The PBS spectra were subtracted to correct the background fluorescence.

### 2.3. FT-IR Spectroscopic Measurements

A Thermo-Nicolet 6700 FT-IR spectrometer (ThermoFisher Scientific, Waltham, MA, USA) was employed to measure infrared spectra. The attenuated total reflectance (ATR) method was applied to collect all spectra at a resolution of 4 cm^–1^ and 64 scans. The infrared spectra of HSA with and without PFCAs were recorded at 1800–1400 cm^–1^. The molar ratio of each PFCA to HSA was 1:1 [27]. The spectra of PBS and each PFCA were determined at the same condition and subtracted to observe the infrared spectra of the sample solution. The second derivative was used in this range to assess the number, position, and width of component bands. The best Gaussian-shaped curves were obtained through curve fitting of the Galactic peak based on these parameters. After identification, the area of each band of HSA with a representative secondary structure was determined [28].

### 2.4. Molecular Docking Study

The crystal structure of HSA (PDB ID: 1H9Z) was downloaded from the RCSB Protein Data Bank (http://www.rcsb.org). PFBA (ZINC ID: 3861259), PFHxA (ZINC ID: 38141478), PFOA (ZINC ID: 6844606), and PFDA (ZINC ID: 6845007) were obtained from the Zinc (http://zinc.docking.org/) site as 3D mol2 text [29]. The Scripps Research Institute’s AutoDock Vina (http://vina.scripps.edu/) and MGLTools (http://mgltools.scripps.edu/) sites were used for docking calculations [30]. A grid of 40 Å × 40 Å × 40 Å spacing was calculated. The docking with the grid box centered at (32.033, 10.568, 4.112) in HSA indicates binding at site I for PFCAs [23]. The output of AutoDock Vina was rendered using Discovery Studio 3.5 (Discovery Studio 3.5, Accelrys, Inc., San Diego, CA, USA).

## 3. Results and Discussion

### 3.1. The Binding Mechanism between PFCAs and HSA

**Figure 1:**
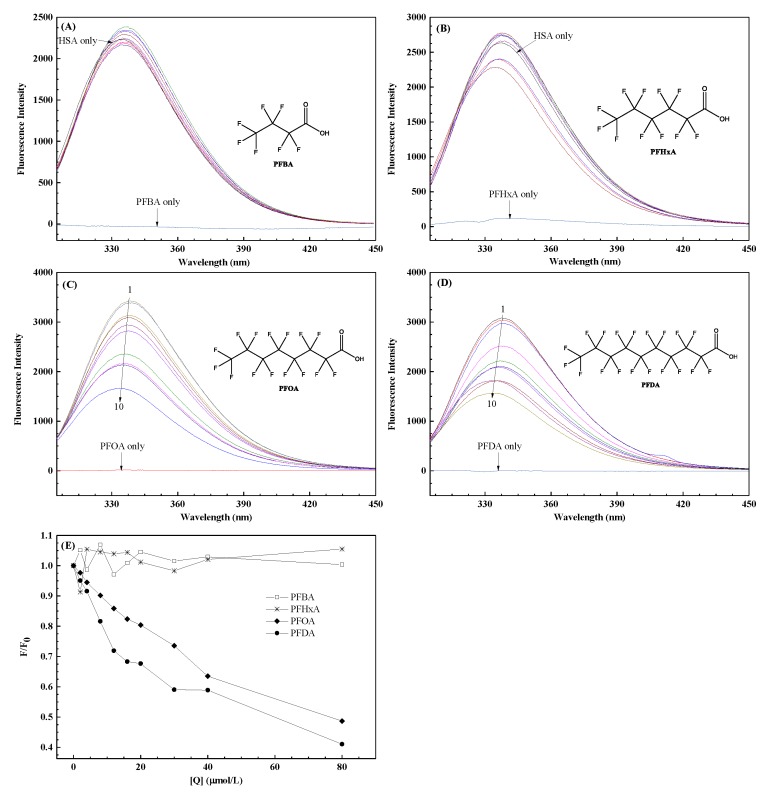
The fluorescence quenching spectra of human serum albumin (HSA) by perfluorobutanoic acid (PFBA) (**A**), perfluorohexanoic acid (PFHxA) (**B**), perfluorooctanoic acid (PFOA) (**C**) and perfluorodecanoic acid (PFDA) (**D**) at 296 K, *λ*_ex_ = 295 nm; the inset corresponds to the molecular structure of PFCAs; (**E**) Normalized fluorescence intensity of HSA with different PFCA concentrations. [PFCAs] (×10^–6^ mol/L) 1–10: 0, 2, 4, 8, 12, 16, 20, 30, 40 and 80. [HSA] = 2 × 10^–6^ mol/L; Buffer: PBS, pH = 7.4.

Figure 1 illustrates the fluorescence spectra of HSA at 296 K in a series of PFCA concentrations. As shown in Figure 1A,B, fluorescence intensities change to a small extent by adding PFBA and PFHxA, and there is a slight shift in the emission wavelength. On the contrary, the spectra of the PFOA–HSA and PFDA–HSA systems show remarkable fluorescence quenching with increasing concentration (Figure 1C,D). At the same time, as the PFOA/PFDA concentration increases, the fluorescence intensity of HSA decreases being accompanied by a blue shift, suggesting that Trp transits from a more polar environment to a less polar environment [31].

To make the trend clearer, Figure 1E displays the normalized fluorescence *F*/*F*_0_ of spectrum versus PFCA concentration, where *F*_0_ and *F* are the fluorescence intensity of HSA before and after adding PFCAs, respectively. When comparing the general trend of four curves in Figure 1E, the slight fluctuation of the PFBA/PFHxA curve shows that PFBA and PFHxA have little effect on the fluorescence intensity of HSA. However, PFOA and PFDA obviously decrease the fluorescence intensity of HSA in the studied concentrations. In addition, based on the slopes of PFOA and PFDA curves, the combination of PFDA and HSA is stronger than PFOA. That is to say, PFDA is more likely to cause a health hazard than PFOA. Our further study of the fluorescence quenching mechanism was limited to PFOA and PFDA.

In order to further study the quenching process of PFCAs, fluorescence assays were conducted at different temperatures. The reduction of fluorescence quenching intensity is normally defined by the famous Stern–Volmer formula [31]:

(1) $$\frac{F_{0}}{F}=1+K_{\mathrm{SV}}[Q]=1+k_{q}\tau_{0}[Q]$$

where *F*_0_ and *F* are the fluorescence intensities in the absence and presence of a quenching agent, respectively. *K*_SV_ is the Stern–Volmer quenching constant calculated using a linear regression curve of *F*_0_/*F* versus [*Q*]; *k*_q_ represents the quenching rate constant of biological molecules; *τ*_0_ is the average fluorescence lifetime without a quenching agent, and it is considered to be 10^–8^ s in general [31]; and [Q] is the concentration of the quenching agent. Under normal conditions, the maximum dynamic *k*_q_ of various quenching agents is 2.0 × 10^10^ L/mol·s [31].

**Figure 2:**
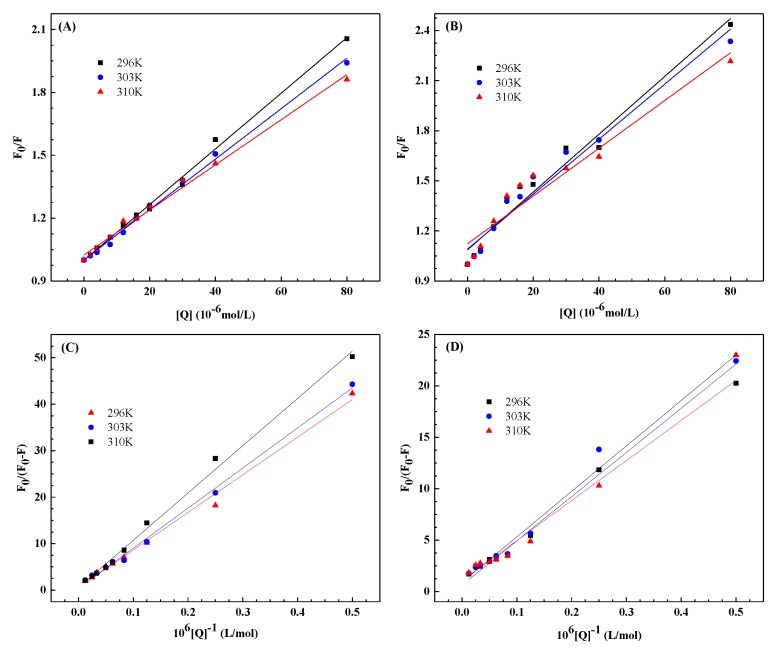
(1) Stern–Volmer plots of the PFOA–HSA (**A**) and PFDA–HSA (**B**) systems at different temperatures; (2) The modified Stern–Volmer plots of the PFOA–HSA (**C**) and PFDA–HSA (**D**) systems at different temperatures.

Figure 2A,B shows the Stern–Volmer diagrams of HSA quenched by PFCAs at different temperatures (296, 303, and 310 K). Table 1 lists the quenching constant calculated at the corresponding temperature. The results indicate that *K*_SV_ is inversely proportional to temperature with *k*_q_ higher than 2.0 × 10^10^ L/mol·s, meaning that the interaction between PFOA/PFDA and HSA is complicated by formation not caused by dynamic collisions.

The data were analyzed for the static quenching procedure following the modified Stern–Volmer formula [32]:

(2) $$\frac{F_{0}}{\Delta F}=\frac{F_{0}}{F_{0}-F}=\frac{1}{f_{a}K_{a}}\frac{1}{\left[Q\right]}+\frac{1}{f_{a}}$$

where *f*_a_ represents the fraction of accessible fluorescence, and *K*_a_ represents the effective quenching constant of accessible fluorophores. The dependence of *F*_0_/Δ*F* on the reciprocal of the quencher concentration [*Q*] is linear, and its slope equals to (*f*_a_*K*_a_)^−1^; *f*_a_^–1^ is fixed on the ordinate; and *K*_a_ is the quotient of *f*_a_^−1^ and (*f*_a_*K*_a_)^−1^. Figure 2C,D shows linear graphs at different temperatures on the basis of formula (2). Table 2 displays the corresponding values of *K*_a_. The tendency of *K*_a_ to decrease with increasing temperature is consistent with the temperature dependence of *K*_SV_ as mentioned above in accordance with the static quenching mechanism. A decrease in the *K*_a_ value with temperature indicates that the binding reaction of PFOA/PFDA with HSA is exothermic [33]. The negative enthalpy change (Δ*H*) determined in the next section verifies this. Moreover, the *K*_a_ of PFDA–HSA is far higher than that of PFOA–HSA, suggesting that the affinity of PFDA with 10 carbons is higher than that of PFOA with 8 carbons, which is consistent with the results in Figure 1E; this discovery is supported by the fluorescence study conducted by Qin et al. [26]. As suggested by Cheng and Ng [34], the relationship between carbon chain length and binding affinity was mainly caused by the van der Waals interaction energy and entropy change during binding, both of which were closely related to carbon chain length. The results in Table 2 indicate as well that the binding constants of PFOA/PFDA to HSA are moderate, suggesting that PFOA/PFDA can be stored and transported in vivo by HSA [35].

### 3.2. The Characterization of Binding Force between PFOA/PFDA and HSA

**Figure 3:**
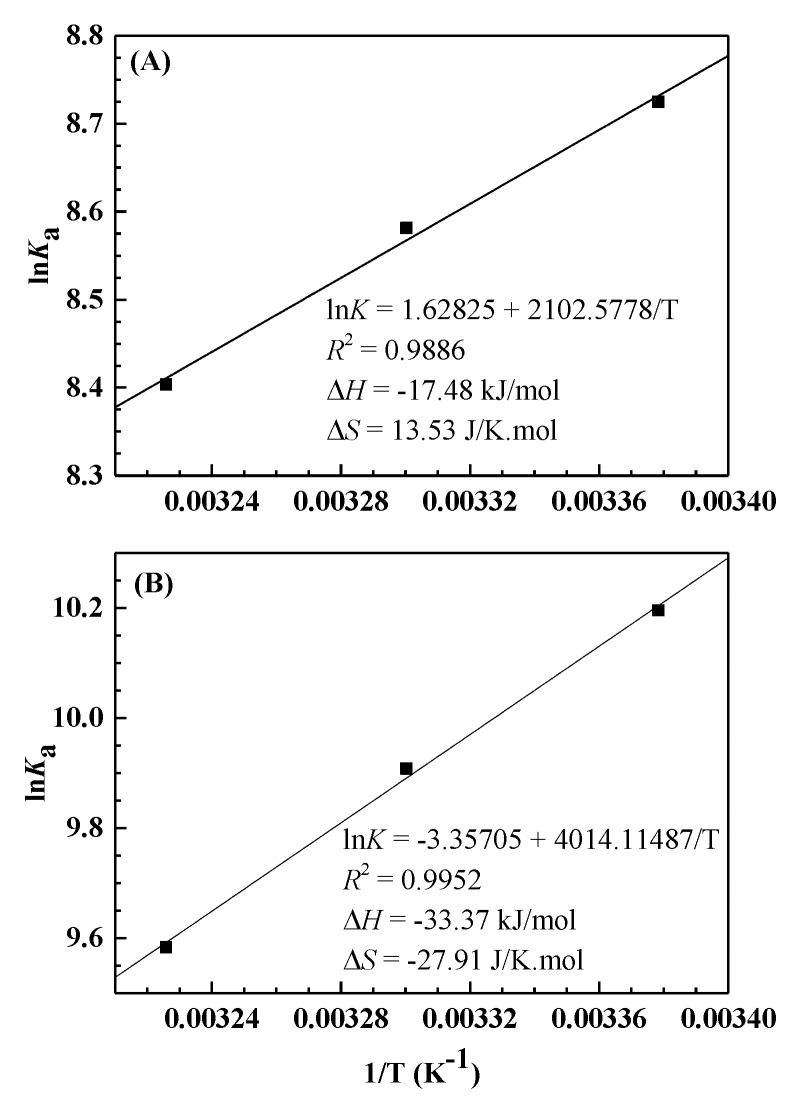
Van ’t Hoff plots of the PFOA–HSA (**A**) and PFDA–HSA (**B**) systems at different temperatures.

Generally, interactions between drugs and proteins can contain electrostatic interaction, van der Waals interaction, hydrophobic force, and hydrogen bonding. To clarify the binding of PFOA/PFDA to HSA, the thermodynamic parameters were determined based on the van ’t Hoff formula:

(3) $$\ln K=-\frac{\Delta H}{RT}+\frac{\Delta S}{R}$$

where *K* is similar to the effective quenching constant *K*_a_, and *R* represents the gas constant. As we can see in Figure 3, there is a good linear relationship between ln*K* and 1/*T*, which means that the enthalpy change (Δ*H*) is constant. Then the free energy change (Δ*G*) is obtained according to the following formula:

(4) ΔG=ΔH−TΔS=−RTlnK

Table 2 presents the corresponding results. According to the thermodynamic laws for determining the binding type summed up by Ross and Subramanian [36], the positive Δ*S* and negative Δ*H* for the PFOA–HSA complex demonstrate that electrostatic interaction is the major force in the binding process, while the negative Δ*S* and Δ*H* for PFDA–HSA complex indicate that van der Waals force and hydrogen bonding are dominant in binding. The negative Δ*G* for both PFOA and PFDA indicates a spontaneous binding process.

### 3.3. The Conformational Changes of HSA Induced by PFCAs

**Figure 4:**
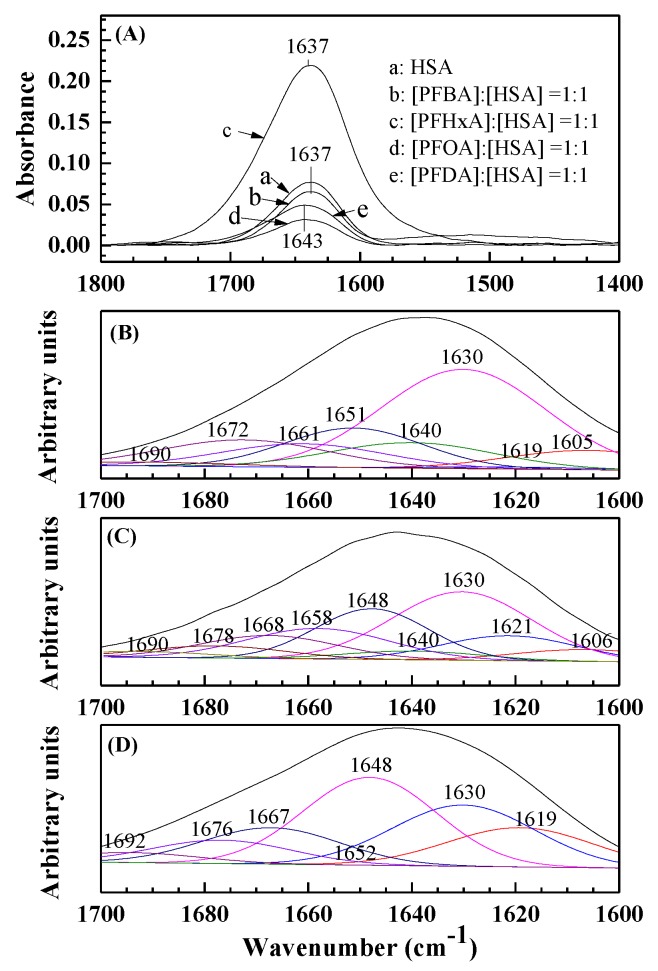
(1) The FT-IR spectra (**A**) of free HSA (a), difference spectra [(PFBA–HSA) – PFBA solution] ([PFBA]:[HSA] = 1:1) (b), difference spectra [(PFHxA–HSA) – PFHxA solution] ([PFHxA]:[HSA] = 1:1) (c), difference spectra [(PFOA–HSA) – PFOA solution] ([PFOA]:[HSA] = 1:1) (d), and difference spectra [(PFDA–HSA) – PFDA solution] ([PFDA]:[HSA] = 1:1) (e) in a pH 7.4 buffer solution in the region of 1800–1400 cm^−1^; (2) The curve-fit amide I (1700–1600 cm^−1^) region with the secondary structure determination of the free HSA (**B**), PFOA–HSA complex (**C**), and the PFDA–HSA complex (**D**) in a pH 7.4 buffer solution. [HSA] = 2 × 10^–6^ mol/L.

The infrared spectrum of proteins shows many amide bands, which are different vibrations of peptides. Among them, amide I bands (1700–1600 cm^−1^, primarily C=O stretching) and amide II bands (1600–1500 cm^−1^, C-N stretching coupled with N-H bending) are related to the secondary structure of a protein, whereas amide I bands are more susceptible to changes in the secondary structure of a protein than amide II bands [37]. The infrared spectra of free HSA and the difference spectra of the PFCA–HSA complex are exhibited in Figure 4A. The peak position of amide I in HSA shifted from 1637 to 1643 cm^-1^, pointing out that the secondary structure of HSA changed due to the binding of PFOA/PFDA to HSA [38]. Nevertheless, the peak positions of amide I in PFBA–HSA and PFHxA–HSA systems do not change, suggesting that PFBA and PFHxA have little influence on the HSA structure.

According to Barth [37], spectral ranges 1660-1700 cm^−1^, 1650-1659 cm^−1^, 1640-1650 cm^−1^, and 1610-1640 cm^−1^ are allocated to β-turn, α-helix, random coil, and β-sheet, respectively. Figure 4B–D shows the curve-fitted infrared spectra of HSA and its components, assignments and compositions in order to compare the structure of HSA in the absence and presence of PFCAs more meaningfully. The percentage of each secondary structure of HSA can be obtained using the integrated area of each component band in amide I (Table 3). The sharp decrease in the β-sheet and α-helix conformations of the PFOA–HSA system and the PFDA–HSA system associated with the free HSA structure is in agreement with the assumption that the interaction of PFOA/PFDA with HSA altered the secondary structure of HSA and resulted in a partial protein destabilization [39]. However, Wu et al. [40] reported that PFOA binding decreased the β-sheet content of HSA, but increased the α-helix content by 15%. This inconsistence may be due to the different buffers used [41].

### 3.4. Molecular Modeling Results

**Figure 5:**
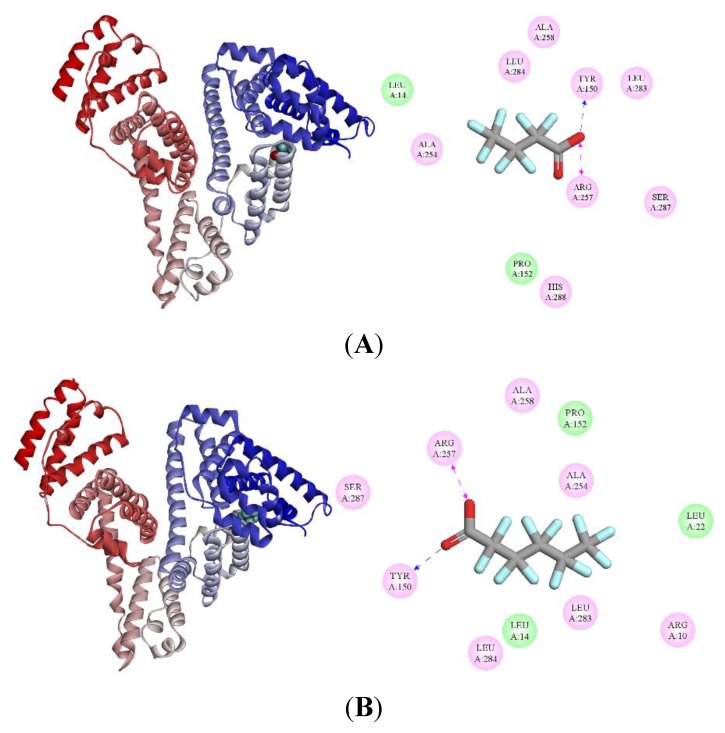

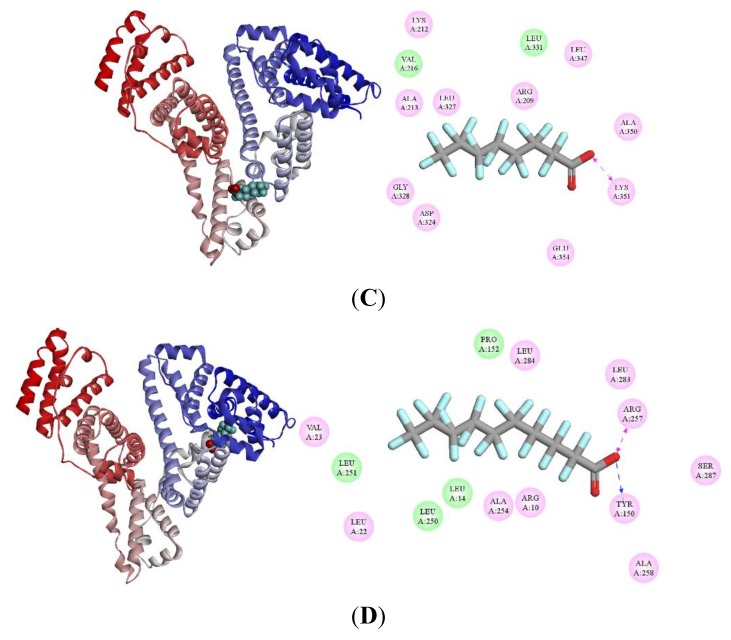
Left: Binding site of PFCAs on HSA (PDB ID: 1H9Z). HSA is shown in cartoon. PFCAs are represented using spheres. Right: 2D ligand interaction diagram of PFCAs with HSA using Discovery Studio with the essential amino acid residues at the binding site tagged in circles. Pink circles show the amino acids that participate in electrostatic interactions, and green circles show the amino acids that participate in van der Waals interactions. Hydrogen-bond interactions with amino acid side-chains (blue dashed arrow) and charge interactions (pink dashed arrow) are indicated. (**A**) PFBA–HSA; (**B**) PFHxA–HSA; (**C**) PFOA–HSA; and (**D**) PFDA–HSA.

To study the binding affinities of PFCAs to HSA systemically, molecular modeling was applied by setting a simulation box to site I. The best energy-ranked result as shown in Figure 5 (left) demonstrated that PFCAs bind within the pocket of sub-domain IIA of HSA (site I), which is in accordance with the results obtained by Salvalaglio et al. [42]. The calculated lowest binding free energies (ΔG) are −24.28, −30.55, −34.74, and −40.18 kJ/mol for PFBA–HSA, PFHxA–HSA, PFOA–HSA, and PFDA–HSA, respectively, which indicates that PFCAs with a longer carbon chain have a lower ΔG suggesting a stronger binding affinity to HSA. This is in line with the fluorescence research. As described above, the van der Waals interaction energy and entropy change during binding related to carbon chain length could explain this phenomenon [34].

Figure 5 (right) shows 2D models of binding of PFCAs to HSA. The amino acid residues (AARs) around site I which take part in the interaction between PFCAs and HSA are composed of Arg 10, Leu 14, Leu 22, Val 23, Try 150, Pro 152, Arg 209, Lys 212, Ala 213, Val 216, Leu 250, Leu 251, Ala 254, Arg 257, Ala 258, Leu 283, Leu 284, Ser 287, His 288, Asp 324, Leu 327, Gly 328, Leu 331, Leu 347, Ala 350, Lys 351, and Glu 354. More than 10 AARs are lying around site I, and the essential driving forces of PFOA binding to this site are electrostatic interaction and van der Waals force, while van der Waals interaction, hydrogen bond (PFDA with Tyr 150), and electrostatic interaction are major driving forces in the combination of PFDA and HSA. The results are in line with the experimental study.

## 4. Conclusions

In summary, the interaction mechanism of PFCAs with different carbon chain length with HSA was studied at the molecular level by spectroscopy. PFCAs with longer carbon chains caused stronger fluorescence quenching. According to the fluorescence spectra and FT-IR data, it was found that PFBA and PFHxA had little effect on HSA. PFOA and PFDA interacted with HSA by means of a static quenching mechanism to spontaneously form a medium-intensity complex. Thermodynamic parameters indicated that the interaction force of the PFOA–HSA complex is electrostatic interaction, while van der Waals interaction and hydrogen bond play an important role in the combination of PFDA and HSA. Additionally, PFDA was found to have a stronger binding capacity than PFOA, which was supported by molecular docking. Moreover, infrared spectroscopy confirmed that the microenvironment and conformation of HSA were changed by PFOA/PFDA, and the α-helix and β-sheet conformation decreased sharply. Our results indicate that the carbon chain length of PFCAs has a great influence on their binding affinity. These data can provide certain quantitative information for future studies of molecular toxicology of PFCAs with different carbon chain length.

## Figures and Tables

**Table 1:** Stern–Volmer quenching constants of the PFCA–HSA system at different temperatures.

PFCA	*T* (K)	*K*_SV_ (×10^4^ L/mol)	*k*_q_ (×10^12^ L/mol·s)	*R* ^a^
PFOA	296	1.328 ± 0.003	1.328 ± 0.003	0.9978
	303	1.203 ± 0.003	1.203 ± 0.003	0.9981
	310	1.076 ± 0.001	1.076 ± 0.001	0.9964
PFDA	296	1.731 ± 0.001	1.731 ± 0.001	0.9836
	303	1.647 ± 0.002	1.647 ± 0.002	0.9814
	310	1.431 ± 0.001	1.431 ± 0.001	0.9648

^a^*R* is the correlation coefficient for Stern–Volmer plots.

**Table 2:** Modified Stern–Volmer association constants *K*_a_ and relative thermodynamic parameters of the PFCA–HSA system at different temperatures.

PFCA	*T* (K)	*K*_a_ (×10^4^ L/mol)	*R* ^a^	Δ*H* (kJ/mol)	Δ*S* (J/mol·K)	Δ*G* (kJ/mol)	*R* ^b^
PFOA	296	0.6153	0.9965	−17.48 ± 0.33	13.53 ± 0.17	−21.49 ± 0.03	0.9971
	303	0.5332	0.9975			−21.58 ± 0.02	
	310	0.4463	0.9984			−21.67 ± 0.04	
PFDA	296	2.6788	0.9962	−33.37 ± 0.46	−27.91 ± 0.29	−25.11 ± 0.03	0.9988
	303	2.0093	0.9929			−24.92 ± 0.04	
	310	1.4514	0.9926			−24.72 ± 0.02	

^a^*R* is the correlation coefficient for modified Stern–Volmer plots; ^b^ *R* is the correlation coefficient for van ’t Hoff plots.

**Table 3:** Secondary structure analysis (infrared spectra) for free HSA and its drug complexes at pH 7.4.

Complex	β-Sheet	Random Coil	α-Helix	β-Turn
Free HSA	51.38	10.99	14.19	23.44
PFOA–HSA	46.28	21.96	9.82	21.94
PFDA–HSA	39.37	32.65	1.02	26.95
